# Separation of Zn(II), Cr(III), and Ni(II) Ions Using the Polymer Inclusion Membranes Containing Acetylacetone Derivative as the Carrier

**DOI:** 10.3390/membranes10050088

**Published:** 2020-04-30

**Authors:** Elzbieta Radzyminska-Lenarcik, Ilona Pyszka, Malgorzata Ulewicz

**Affiliations:** 1 Faculty of Chemical Technology and Engineering, UTP University of Science and Technology, Seminaryjna 3, PL 85-326 Bydgoszcz, Poland; ilona.pyszka@utp.edu.pl; 2 Faculty of Civil Engineering, Czestochowa University of Technology, Dabrowskiego 69 Street, PL 42-201 Czestochowa, Poland; malgorzata.ulewicz@pcz.pl

**Keywords:** polymer inclusion membrane, metal separation, acetylacetone derivatives

## Abstract

Polymer inclusion membranes (PIMs) doped with ethylenodiamino-bis-acetylacetone as fixed carrier was applied for the investigation of the facilitated transport of Zn(II), Cr(III), and Ni(II) ions from an aqueous nitrate feed phase (c_M_ = 0.001 mol/dm^3^). The optimal membrane composition (amount of carrier and *o*-NPPE-plasticizer) was determined. For the optimal polymer inclusion membranes doped with ethylenodiamino-bis-acetylacetone, the following patterns of transport selectivity were found: Zn(II) > Cr(III) > Ni(II). The initial flux of Zn(II), Cr(III), and Ni(II) ions was 6.37 µmol/m^2^∙s, 5.53 µmol/m^2^∙s, and 0.40 µmol/m^2^∙s, respectively. The selectivity coefficients equal to 1.2 and 15.9 were found for Zn(II)/Cr(III) and Zn(II)/Ni(II), respectively. After 24-h transport, the recovery factor of Zn(II), Cr(III), and Ni(II) were 90%, 65%, and 6%, respectively. The polymer inclusion membranes doped with ethylenodiamino-bis-acetylacetone were characterized by scanning electron microscopy and non-contact atomic force microscopy. The influence of membrane morphology on transport process was discussed.

## 1. Introduction

Chromium(III), zinc(II), and nickel(II) are used in various fields of technology. Among other purposes, they are used to protect materials against corrosion and to give them a decorative appearance. During galvanic waste treatment, metal ions are precipitated, and this process generates heavy metal bearing sludge. It is categorized as hazardous waste due to the presence of various heavy metal ions (Cu(II), Ni(II), Cr(III), Cd(II), Zn(II)) [1]. Heavy metal ions are permanently present in the Earth’s crust. However, their concentrations increased with industrial applications, and their contents changed [2]. Galvanizing plants contain significant amounts of heavy metal ions, which pose a great threat to human and animal life and the environment.

The use of membrane technology is now very popular for the separation of liquid–liquid mixtures. Current liquid membrane separation technologies are not expensive, because energy consumption is low and metal ion carriers can be recovered for reuse. In liquid membrane technologies, energy consumption is lower than membrane technologies based on pressure-driven alternatives such as, for example osmosis, nanofiltration, or microfiltration. The technology of liquid membranes is more ecological and economical [3,4,5].

The most commonly used liquid membranes are supported or immobilized liquid membranes (SLM) [6]. Polymer inclusion membranes (PIM) are a kind of liquid membranes that visually resemble SLM but show greater stability [4,5,7,8]. PIM is executed from support (polymer), ion extractant (carrier), and plasticizer using a volatile solvent. Cellulose triacetate (CTA) [9,10,11,12,13] or polyvinyl chloride (PVC) [14,15,16,17,18,19] is generally used as the support to maintain mechanical strength to the membrane while the carrier forms complexes with the transferred ions to ensure the selectivity of their separation. However, most of the commercial extractants used so far as non-ferrous metal ion carriers in the process of transport across LMs do not ensure satisfactory selectivity towards a number of metal ions. Therefore, it seemed justified to search for new selective carriers enabling the separation of metal ions from aqueous solutions. However, different ion carriers demonstrate different transport efficiencies [20]. To date, research on PIMs for removing various heavy metal ions such as As(V) [21,22], Cd(II) [13,23,24,25], Cu(II) [12,25,26,27], Mn(II) [28], Ag(I) [12,27], Pb(II) [29,30,31], Cr(III,IV) [10,24,32,33], and Zn(II) [9,13,24,27,34] have been successful and promising. Membrane technologies have become a very important alternative to conventional processes used for wastewater treatment, separation of metal ions, or concentration of metals [8,35]. The selective transport of metal ions through supported (SLMs) and polymer inclusion (PIMs) liquid membranes has been widely studied [8,26,36,37,38,39,40,41,42,43,44,45]. Their high selectivity, high diffusion rates, and the ability to concentrate ions and stability have made them particularly useful technology [4,8].

The main purpose of this work was to application a new compound ethylenodiamino-bis-acetylacetone as a carrier in PIMs and to check its usefulness in metal separation. Moreover, the aim of this work included the investigation of the physical properties of CTA-based membranes on their efficiency in the separation of Zn(II)-Cr(III)-Ni(II) ions during transport and stability this process.

## 2. Experimental

### 2.1. Reagents and Apparatus

The following reagents were used for the investigation: Zn(NO_3_)_2_·H_2_O (POCh, Gliwice, Poland), Cr(NO_3_)_3_·9H_2_O (POCh, Gliwice, Poland), Ni(NO_3_)_2_·6H_2_O (POCh, Gliwice, Poland), tetramethylammonium hydroxide (N(CH_3_)^4+^·OH^−^) (POCh, Gliwice, Poland), cellulose triacetate (CTA) (Fluka, Switzerland), *o*-nitrophenyl pentyl ether (*o*-NPPE) (Fluka, Switzerland), and dichloromethane (Fluka, Switzerland). All reagents used were of analytical grade. standard buffer solutions (pH = 7.00 ± 0.01, pH = 9.21 ± 0.01) (Radiometer, Copenhagen, Denmark) were also used for pH-meter calibration.

**Figure 1:**
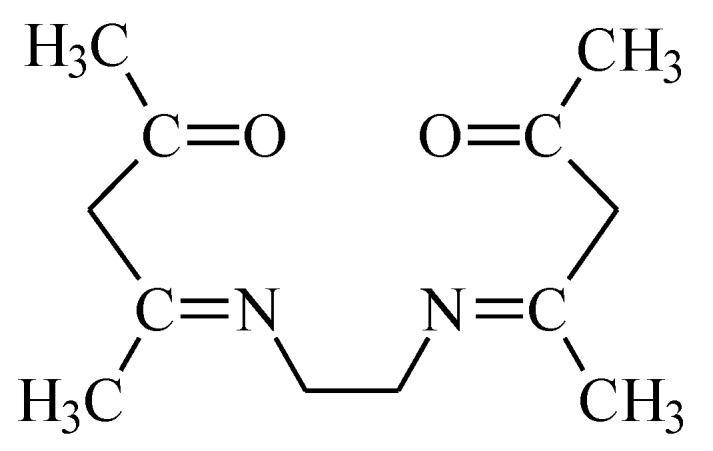
The chemical formula of ethylenodiamino-bis-acetylacetone (EDAB-acac).

The reagent ethylenodiamino-bis-acetylacetone (EDAB-acac) (Figure 1) was synthesized using methods for obtaining Schiff bases [46] according to Equation (1). The melting point of the obtained compound was 110–111 °C.


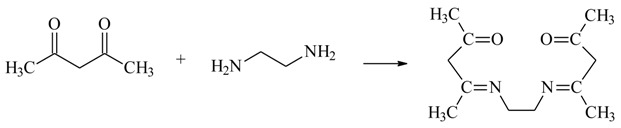
(1)

The ethylenodiamino-bis-acetylacetone (EDAB-acac) structure was confirmed by ^1^H NMR (400 MHz), ^13^C NMR (100.6 MHz), and ^15^N NMR (40 MHz), spectroscopy (Brucker, Germany). ^1^H NMR, CDCl_3_, δ(ppm): 1.9116 (s, 6H, C–CH_3_), 2.0033 (s, 6H, C–CH_3_), 3.4253 (s, 4H, C–CH_2_–C), 4.996 (t, 4H, CH_2_–N^1^); ^13^C NMR, CDCl_3_, δ(ppm): 18.6761, 18.6998, 28.8661, 28.9183, 43.4035, 43.5173, 162.6401, 162.9292, 195.4509, 195.4990; ^15^N NMR CDCl_3_, δ(ppm): 106.56, 154.51.

### 2.2. Polymer Inclusion Membrane Preparation

PIMs were obtained by a commonly used pouring method [13,23,26,28,39,44,45]. The CTA, *o*-NPPE, and EDAB-acac solution in the organic solvent was slowly evaporated. The wet membrane contained 2.7 cm^3^ *o*-NPPE/1g CTA, and 0.2–1.0 mol/dm^3^ of EDAB-acac based on plasticizer.

Membrane thickness was measured using a digital micrometer (Panametrics^®^ Magna-Mike^®^ 8500 (San Diego, CA, USA)) with an accuracy of 0.1 µm. It was found that their thickness before and after the transport process is the same. A surface characterization study of the membranes was performed using an Atomic-force MultiMode Scanning Probe Microscope IIIa (AFM) (Digital Instruments Vecco Metrology Group, Santa Barbara, CA, USA) according to the procedure described in other papers [43,44,45,47].

### 2.3. Transport Studies

Transport experiments were carried out in the system described in earlier papers [13,23,26,28,39,43,44,45,47] at 20 ± 0.2 °C. The feed phase was an aqueous solution of metal salts with pH = 7.8 maintained by tetramethylammonium hydroxide and controlled by pH-meter (pH meter, CX-731 Elmetron, Poland with a combination pH electrode HER-126, Hydromet, Poland). The receiving phase was deionized water, pH = 6.8.

At the receiving phase, metal ions concentrations were measured at appropriate intervals by atomic absorption spectroscopy (AAS 240FS Spectrometer, Agilent, Santa Clara, CA, USA).

### 2.4. Applied Calculations Related to the Parameters Characterizing Transport

According to Danesi [48], the kinetics of transport of metal ions across membranes in relation to their concentration (c) can be described by the first order equation:

(2) $$\ln\left(\frac{c}{c_{i}}\right)=-\mathrm{kt}$$

where *c* is the metal ions concentration in the feed phase at a given time (mol/dm^3^), *c_i_* is the initial metal ions concentration in the feed phase, *k* is the rate constant (s^−1^), and *t* is the time of transport (s).

The characteristic parameters for the metal ions transport across membranes are summarized in Table 1.

## 3. Results and Discussion

### 3.1. Membranes Characterization

**Figure 2:**
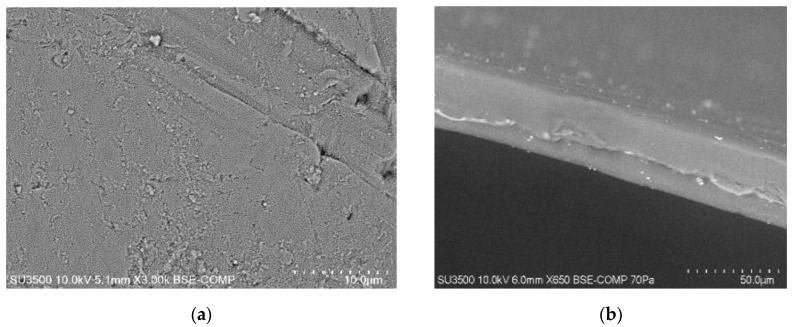
SEM picture of PIMs doped with ethylenediamine-bis-acetylacetone as: Front view (**a**), side view (**b**).

The selectivity of metal ion transport through membranes depends primarily on the physico-chemical properties of polymeric inclusion membranes [13,23,26,28,39,42,43,44,47]. As demonstrated in papers [48,49], the porosity as well as roughness of the membranes is determined mainly by the kind and concentration of the ion carrier. Therefore, in the first stage of research, newly synthesized polymeric inclusion membranes were subjected to microscopic tests. The SEM pictures (Figure 2) showed that all membranes demonstrated dense and homogeneous structures. In addition, the membrane surface roughness was visible in the pictures. A carrier (for example ethylenodiamino-bis-acetylacetone molecules) could crystallise in the membrane and migrated to the membrane surface, bring about its porosity and roughness. However, as seen in Figure 2, the distribution of the EDAB-acac in the investigated membrane, after the evaporation of dichloromethane, is homogeneous on its entire surface.

**Figure 3:**
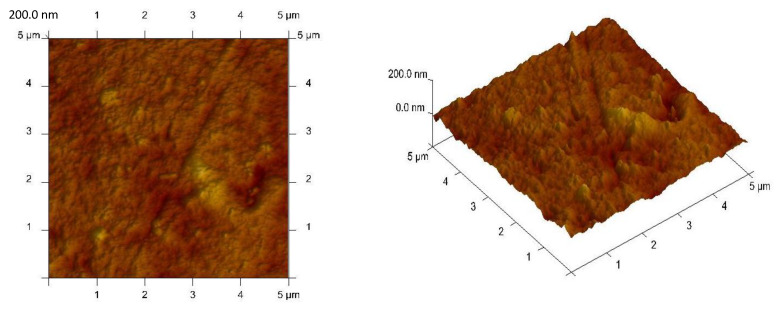
AFM 2D and 3D picture of PIMs doped with ethylenediamine-bis-acetylacetone at a 0.8 mol/dm^3^ concentration.

Figure 3 shows an atomic force microscopy (AMF) picture of PIM with the ethylenediamine-bis-acetylacetone in a two- and three-dimensional form in the 5.0 × 5.0 μm^2^. In the image of the PIMs (Figure 3) sample clearly visible are elongated pores called also “cavity channels” (darker regions). The morphology of this membrane surface is associated to the crystallinity of the CTA. An extensive pore (5–25 nm in size) is likely to be responsible for the efficiency of the transport process.

The roughness (Rq) and porosity (ε) of the memebranes was characterized using the AFM image processing program NanoScope v.5.12. The R_q_ parameter is the standard deviation of the z values within the box cursor and is calculated according to the procedure described in [43]. The roughness (Rq) and effective pore sizes are shown in Table 2 together with average thickness and the membrane’s tortuosity determined by Equation (3), designated by Wolf and Strieder in paper [42]:

τ = 1 − ln ε(3)

For comparison, the values average thickness, roughness (R_q_) and effective pore sizes of the PVC (polyvinylchloride), DAO (bis-(2-ethylhexyl)adipate), and acetylacetone (acac) derivatives membrane in Table 2 are given [16,50].

Many authors [29,32,43,51,52] have shown that, the microstructure of the membrane has an impact on the transport process. The distribution of pores in CTA membranes is almost uniform (porosity about 50%) [48]. These pores in matrix are filled with a plasticizer, for example *o*-NPPE and the carrier, which crystallizes inside the membrane, causing with the texture of the surface being homogeneous, with different porosities and roughness. The roughness of a CTA membrane achieved by Tor et al. [32] equaled 14 nm. The high roughness (equal 4.40 nm) was obtained for CTA–EDAB–acac membrane in comparison with the PVC membrane doped acac and its hydrocarbon derivatives (Table 1), but for the CTA–EDAB–acac membrane, the pores were smaller in size.

### 3.2. The Effect of Plasticizer Content on Transport of Zn(II) Ions across PIMs with EDAB-acac

Initially, the study involved the transport of zinc(II) ions through PIMs containing EDAB-acac as ionic carriers and with various plasticizer contents. Blank experiments, in the absence of carrier, yielded no significant flux across a PIM containing only the support and the plasticizer. In order to understand the influence of the plasticizer on the transport of zinc ions through the PIM, membranes with different o-NPPE contents were prepared and tested at a temperature of 20 °C.

**Figure 4:**
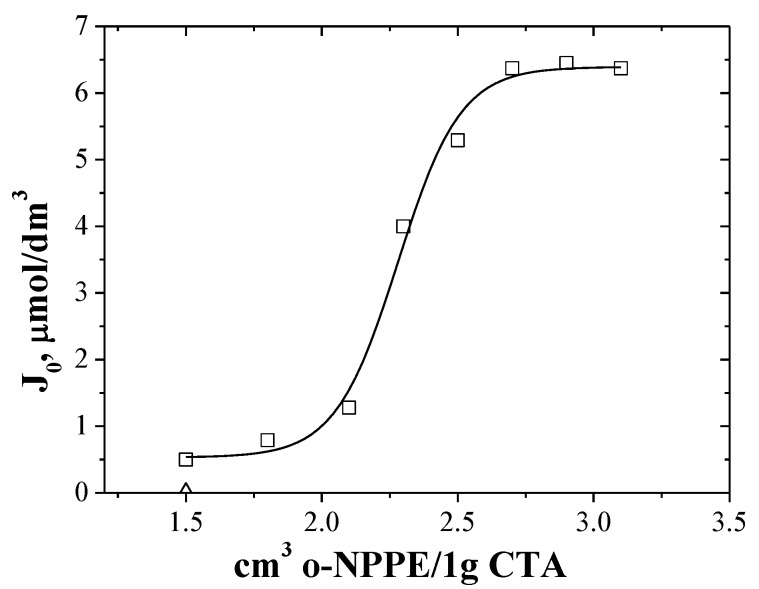
The effect of plasticizer in membranes on the Zn(II) ions initial fluxes during the transport across membrane doped with EDAB-acak.

Based on the data presented in Figure 4, membranes containing 2.7 cm^3^ of the plasticizer per 1 g CTA were selected and prepared for further investigation.

Determining the plasticizer concentration in the membrane is very important because as follows from a few literature reports [24,51], the increase in the concentration of plasticizer in the membrane has a positive effect on the metal ion transfer rate only within a certain range of its concentrations. Gherrou et al. [51] showed that the initial flux of Cu(II) ion transfer through a CTA membrane containing DB18C6 crown ether increases with increasing plasticizer concentration (by NPOE) in the membrane only in the range of 0 from 11 mg per cm^2^ of membrane surface. Whereas Kozlowski et al. [24] shown, that for transport of Cr(VI) the optimal ONPPE plasticizer concentration using TOA (tri-n-octylamine) and TDPNO (4-(1-n-tridecyl)pyridine N-oxide) was 0.8 cm^3^ and 4.0 o-NPPE/1g CTA, respectively. Both low and high plasticizer concentrations in the membrane are undesirable. At a low concentration of the plasticizer in the membrane there is an “anti-softening” effect, and the membrane becomes hard and brittle. On the other hand, too high a concentration of plasticizer in the membrane causes the excess plasticizer to possibly migrate to the water phase, creating at the membrane–water phase interface a barrier to the transport of metal ions.

### 3.3. The Influence of the Carrier Concentration in CTA-o-NPPPE-EDAB-acac Membrane on Transport of Zn(II), Cr(III), and Ni(II) Ions

The effect of carrier concentration on the initial fluxes of Zn(II), Cr(III), and Ni(II) ions during separation of these ions in the membrane process was investigated. The feed phase contained an equimolar mixture of tested ions at a concentration of 0.001 mol/dm^3^ each ion. The concentration of the EDAB-acac in the membrane varied from 0.2 mol/dm^3^ to 1 mol/dm^3^.

The values of initial fluxes for competitive transport of each metal ions across PIMs are shown in Table 3. From the data in Table 3 it follows that the fluxes of all the metal ions rapidly increase with the increase of carrier concentration in the membrane up to a 1.0 mol/dm^3^. The highest initial fluxes of metal ions are found at the 0.8 mol/dm^3^ concentration. Above this concentration, the rate of ion transport is slightly lower. During further testing, membranes with the following composition were used: 2.7 cm^3^ *o*-NPPE/1g CTA and 0.8 mol/dm^3^ of EDAB–acac based on plasticizer.

### 3.4. Separation of Metal Ions from Their Equimolar Solution in Transport across PIMs Doped EDAB-acac

**Figure 5:**
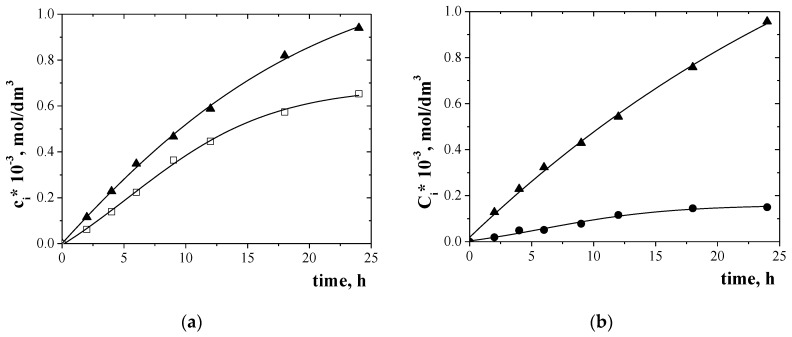
Change in the concentration of Zn (II) (▲) and Cr (III) (□) (**a**), and Zn (II) (▲) and Ni (II) (●) (**b**) in the receiving phase during transport through PIMs doped EDAB-acac. Conditions as in Table 3.

**Figure 6:**
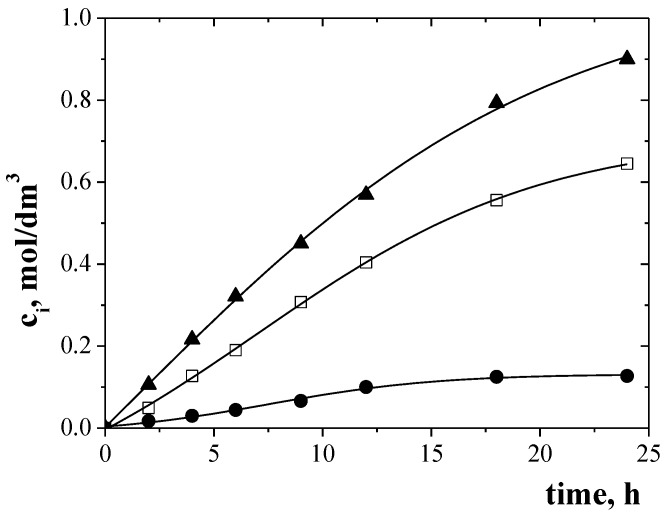
Change in the concentration of metal ions (Zn (II) (▲), Cr (III) (□), Ni (II) (●)) in the receiving phase depending on the transport time. Conditions as in Figure 5.

Change in metal ion concentration in the receiving phase for the transport of Zn(II), Cr(III), and Ni(II) across PIMs doped EDAB-acac is shown in Figure 5 and Figure 6 for binary-component (Figure 5a,b) and three-component (Figure 6) mixture.

Metal ion transport was not registered for over 24 h of continuous process flow. The transport of metal ions across PIMs with EDAB-acac (Figure 5 and Figure 6), according to the model described by Danesi [48], is carried out according to first order kinetics in relation to the concentration of metal ions.

Depending on the pH, the EDAB-acac can form the described Equation (4).


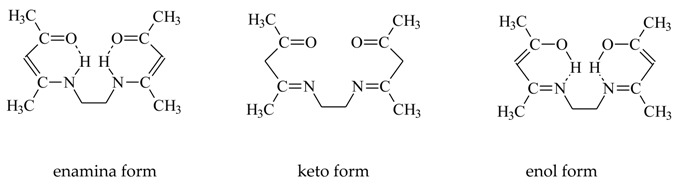
(4)

**Figure 7:**
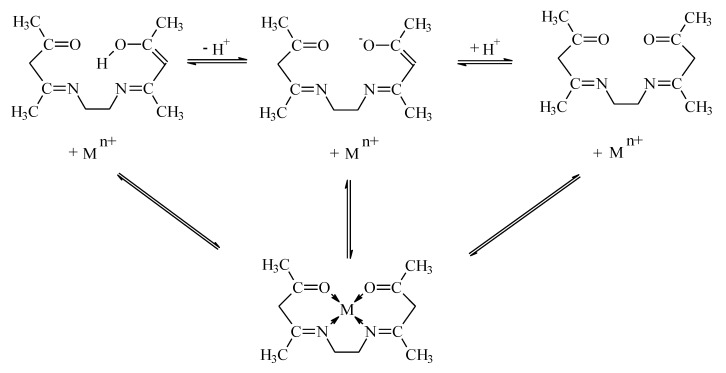
Scheme of EDAB-acac -metal ion complexation.

EDAB-acac with d-block metal cations forming 6-membered chelate complexes with very high stability (Figure 7) [53]. The mechanism of metal ion complexation is very complicated because, according to Miyake [54], both tautomeric forms of β-diketone are reactive towards metal ions. The scheme of EDAB-acac-metal ion complexation is presented in Figure 7.

**Figure 8:**
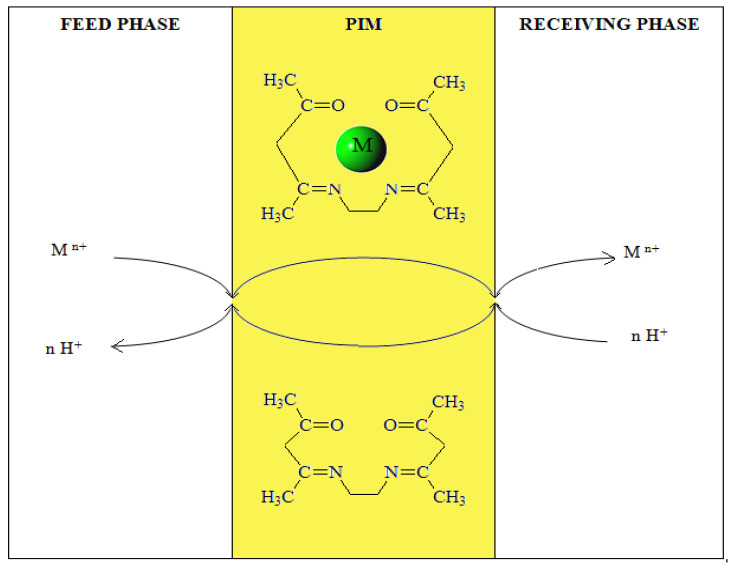
Schematic transport of metal ions across PIMs doped with EDAB-acac.

The transport parameters of investigated metal ions across PIMs doped with EDAB-acac are summarized in Table 4. It follows from the data shown in Table 4 that the initial flux value for Zn(II) ion transport from a unary solution is higher than that from multi-component solutions. In each case the initial fluxes of Ni(II) ions have the lowest values. Selectivity coefficients (S) Zn(II)/Cr(III) and Zn(II)/Ni(II) were equal to 1.4 and 17.6, respectively, for binary solutions and 1.2 and 15.9 for ternary solution. Also a high separation coefficient was obtained for binary Cr(III)-Ni(II) solution (17.6). The Figure 8 presents the proposed mechanism of the Zn(II), Cr(III), and Ni(II) ions transport across PIMs with EDAB-acac.

### 3.5. Recovery of Metal

**Figure 9:**
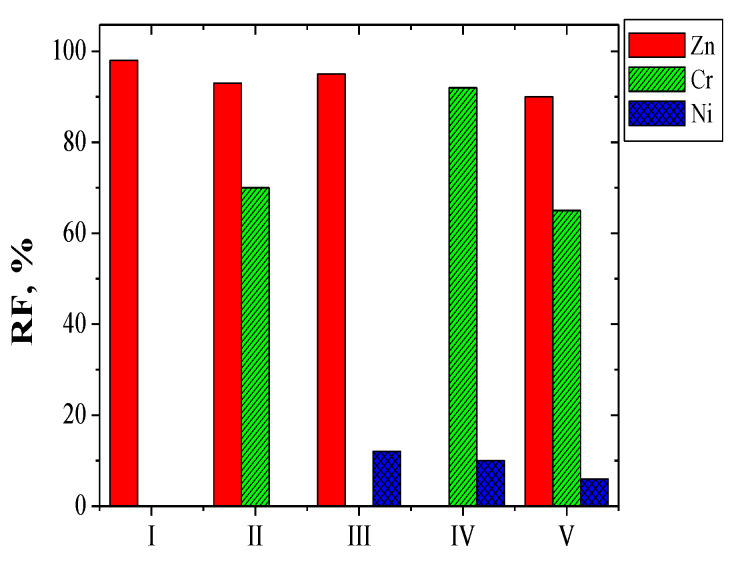
Recovery factor (RF) of zinc, chromium and nickel during transport across PIMs doped with EDAB-acac. Process conditions as in Table 4.

The recovery factors (RF) of investigated metal ions after a 24-h transport are shown in Figure 9. The highest recovery factors (RF) were found for Zn(II) ions for unary solution (98%). The RF values of Zn(II) ions from binary Zn(II)-Cr(III) and Zn(II)-Ni(II) mixture are almost the same (c.a. 93–95%). For binary Cr(III)-Ni(II) solution the RF values for Cr(III) and Ni(II) were 92% and 10%, respectively. In the case of a ternary Zn(II)-Cr(III)-Ni(II) solution, the RF for Zn(II), Cr(III) and Ni(II) were 90%, 65%, and 6%, respectively. The lowest RF values were obtained for Ni(II) ions. Practically, Ni(II) ions were not transported through the PIMs doped with EDAB-acac.

### 3.6. Membrane Diffusion Coefficients of Metal Ions Complexes with EDAB-acac

**Figure 10:**
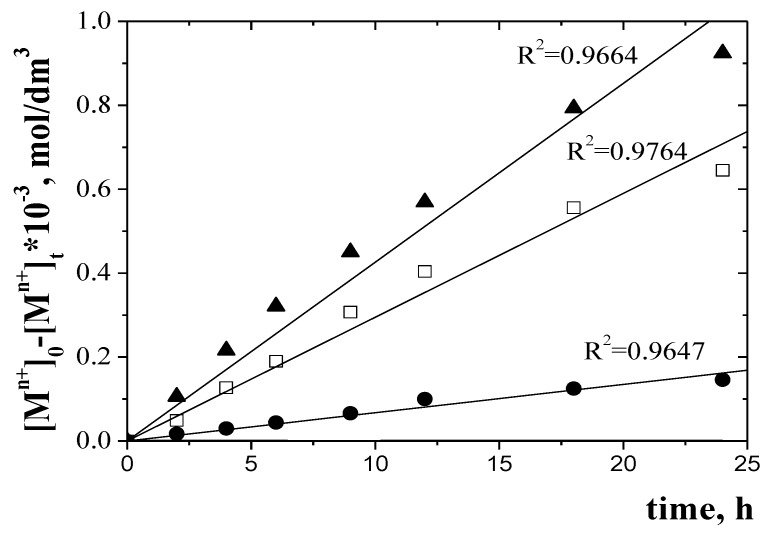
Relationship of [M^n+^]_0_-[M^n+^]_t_ from time for Zn(II) (▲), Cr(III) (□), and Ni(II) (●) transport across PIMs doped with EDAB-acac.

The Figure 10 shows the relationship [M^2+^]_0_-[M^2+^]_t_ = f(t) needed to determine the diffusion coefficients of the investigation metal ions by PIMs doped with EDAB-acac.

The diffusion coefficients characterizing the transport of metal ions were determined using the relationships given by Salazar-Alvarez et al. [29] based on Figure 10.

The diffusion coefficients of metals are summarized in Table 5.

Obtained values of diffusion coefficients are presented in Table 6. Values of diffusion coefficient determined in this study are comparable with those presented in literature data for different membranes are in the range from 10^−12^ to 10^−6^ cm^2^/s and show that the limiting step of the process is the transfer of metal complex across membrane barrier. The value of the diffusion coefficient of M-carrier compounds of 2.362 × 10^−12^–4.061 × 10^−8^ cm^2^/s is smaller than the value reported by Salazar-Alvarez et al. [29]. The values of normalized diffusion coefficients of M^n+^-carrier molecule complexes, obtained in the process of transport across investigated PIM’s from the ternary solution are in the range 6.25 × 10^−13^–1.97 × 10^−9^ cm^2^/s. Thus, the rate of transport of Zn-Cr-Ni ions is determined by the diffusion rate of the complexes M-carrier across the membrane.

### 3.7. Studies of Membrane Stability

The stability of liquid membranes is limited by elution of the conveyor from the membrane to aqueous solutions. As shown in numerous works [7,32,55], the stability of polymer inclusion membranes largely depends on the lipophilicity and surface activity of the conveyor, which undergoes elution from the membrane phase. The stability of the liquid membrane also depends on used a carrier and properly selected organic solvent used as plasticizer.

The recovery rate of Zn (II) transport from Zn (II) -Cr (III) and Zn (II) -Ni (II) solutions across PIM from nitrate solutions to the receiving phase decreases below 5% when using the same membrane four times.

However, the use of the same membrane for the fifth time brings the decrease of the metal ions removal above 10%. Only re-impregnation of the membrane in the carrier (for 24 h) enabled the effective removal of Zn(II) ions from the source phase. The longer lifetimes of a fixed site membrane (FSM) and supported liquid membrane were observed by Gherrou et al. [12] for the transport of Ag(I) with DB18C6 (15 days). In contrast, Ulewicz and Radzyminska-Lenarcik obtained a similar lifetime for the membrane using a 1-hexyl-2-methylimidazole as the carrier in the Cu(II), Zn(II), Co(II), and Ni(II) ion transport process [56].

## 4. Conclusions

Zinc (II) ions can be effectively separated from aqueous solutions of Zn(II), Cr(III), and Ni(II) nitrates by using polymeric inclusion membranes doped with ethylenodiamine-bis-acetylacetone (EDAB-acac). The initial fluxes of metal ions transport decreased in the order: Zn(II) > Cr(III) > Ni(II). The transport rate of the metal ions across PIMs is determined by the diffusion rate of the M(II)-carrier molecule complexes across the membrane. The best result achieved for Zn(II) removal after 24 h was 90% for the ternary Zn(II)-Cr(III)-Ni(II) solution and was almost the same for binary Zn(II)-Cr(III) and Zn(II)-Ni(II) solutions (c.a. 93–95%).

The CTA membrane doped with ethylenodiamino-bis-acetylacetone can also be used to separate Cr(III)-Ni(II) mixture, for which the Cr(III)/Ni(II) separation coefficient was 11.9. The lowest recovery factor values were obtained for Ni(II) ions, which are the slowest transported by this type of membrane. Practically, Ni(II) ions remain in the feeding phase, which can be used to separate Ni(II) from non-ferrous metal ions.

## Figures and Tables

**Table 1:** The characteristic parameters for the ion transport across membrane.

The initial flux (J_i_)	$J_{i}=\frac{V}{A}{\;\mathrm{kc}}_{i}$	V-feed phase volume, m^3^ A-effective area of the membrane, m^2^
The selectivity coefficient (S)	$S=J_{i,M1}/J_{i,M2}$	J_i_-initial flux metal M_1_ or M_2_, µmol/m^2^∙s
The recovery coefficient (RF)	$\mathrm{RF}=\frac{c_{i}-c}{c_{i}}\cdot 100\%$	c-metal ions concentration in the feed phase at a given time, (mol/dm^3^) c_i_-initial metal ions concentration in the feed phase, (mol/dm^3^)

**Table 2:** Parameters characterizing membranes doped with ethylenediamine-bis-acetylacetone (EDAB-acac) and acetylacetone (acac).

Membrane	Average Thickness, µm	Effective Pore Size, µm	Tortuosity	Roughness (R_q_), nm	Ref.
CTA-o-NPPE-0.8 mol/dm^3^ EDAB-acac	30	0.058	2.60	4.40	-
PVC-DAO-60% acac	27	0.063	-	3.55	[16]
PVC-DAO-60% 3-propyl-acac	32	0.060	-	1.76	[50]
PVC-DAO-60% 3-benzyl-acac	33	0.066	-	2.05	[50]

**Table 3:** Permeability coefficients for transport of metal ions through membrane doped with EDAB-acac and recovery factor of metal after 24 h; membrane: 2.7 cm^3^ o-NPPE /1g CTA; feed phase: [M^n+^] = 0.001 mol/dm^3^ each, pH = 7.8, receiving phase: deionized water, pH = 6.8.

Concentration of Carrier, mol/dm^3^	Metal Ions	Initial Flux, J_i_ µmol/m^2^∙s	RF after 24 h, %
0.2	Zn(II)	0.08	14
	Cr(III)	0.05	6
	Ni(II)	0.01	-
0.4	Zn(II)	1.32	25
	Cr(III)	0.72	11
	Ni(II)	0.02	1
0.6	Zn(II)	5.26	77
	Cr(III)	3.40	52
	Ni(II)	0.32	4
0.8	Zn(II)	6.37	90
	Cr(III)	5.53	65
	Ni(II)	0.40	6
1.0	Zn(II)	6.32	83
	Cr(III)	5.49	61
	Ni(II)	0.36	5

**Table 4:** Initial fluxes, order and separation coefficients for competitive transport of Zn(II), Cr(III) and Ni(II) ions through membrane doped with EDAB-acac. Conditions as in Figure 6.

Solutions	Metal Ions	Initial Flux, J_i_ µmol/m^2^∙s	Selectivity Order and Selectivity Coefficients
I	Zn(II)	11.25	-
II	Zn(II) Cr(III)	8.40 5.98	Zn(II) > Cr(III) 1.4
III	Zn(II) Ni(II)	9.16 0.52	Zn(II) > Ni(II) 17.6
IV	Cr(III) Ni(II)	7.27 0.61	Cr(III) > Ni(II) 11.9
V	Zn(II) Cr(III) Ni(II)	6.37 5.53 0.40	Zn(II) > Cr(III) > Ni(II) 1.2 15.9

**Table 5:** List of formulas used in calculations of diffusion coefficient for the ion transport across membrane [29].

the diffusion coefficient (D_o_)	D_o_ = d_o_/Δ_o_	d_o_-the thickness of the membrane (Table 2); Δ_o_ could be seen from Figure 10
the normalized membrane diffusion coefficient (D_o,n_)	D_o,n_= D_o_∙(ε/τ)	

**Table 6:** Values of diffusion coefficients for metal ions transport across PIMs doped with EDAB-acac.

Metal ion	Δ_o_, s/m	D_o_, cm^2^/s	D_o,n_ cm^2^/s
Zn(II)	10^7.33^	4.061 × 10^−8^	1.97 × 10^−9^
Cr(III)	10^6.52^	6.765 × 10^−10^	4.58 × 10^−11^
Ni(II)	10^10.41^	2.362 × 10^−12^	6.25 × 10^−13^
